# Strawberry dietary intervention influences diversity and increases abundances of SCFA-producing bacteria in healthy elderly people

**DOI:** 10.1128/spectrum.01913-24

**Published:** 2025-01-08

**Authors:** Franziska Meiners, Bernd Kreikemeyer, Patrick Newels, Ingmar Zude, Michael Walter, Alexander Hartmann, Daniel Palmer, Georg Fuellen, Israel Barrantes

**Affiliations:** 1 Institut für Biostatistik und Informatik in Medizin und Alternsforschung, Universitätsmedizin Rostock 39071 , Rostock, Germany; 2 Institut für Medizinische Mikrobiologie, Virologie und Hygiene, Universitätsmedizin Rostock, Rostock, Germany; 3 Biovis Diagnostik, Limburg-Offenheim, Germany; 4 Institut für Klinische Chemie und Laboratoriumsmedizin, Universitätsmedizin Rostock 39071 , Rostock, Germany; 5 Conway Institute of Biomolecular and Biomedical Research, School of Medicine, University College Dublin, Dublin, Ireland; Lerner Research Institute, Cleveland, Ohio, USA

**Keywords:** human gut microbiome, dietary intervention, aging

## Abstract

The gut microbiome is amenable to dietary interventions, and polyphenol-rich diets have been shown to enhance abundances of bacteria associated with short-chain fatty acid (SCFA) production. We examined the effects of a strawberry-based intervention on the gut microbiome of 69 healthy elderly German adults. Participants in five groups consumed varying amounts of strawberries, freeze-dried strawberries, and capers in olive oil over 10 weeks as part of a randomized controlled trial. 16S rRNA sequencing was used to analyze differences in microbial composition, diversity, phenotypes, differential abundance, and functional pathways. The intervention group featuring the highest amounts of fresh and freeze-dried strawberries without capers in olive oil (group 4) showed changes in gut microbial diversity and differential abundance that could be linked to improved health. Beta diversity, based on weighted UniFrac distances, increased significantly (*P* = 0.0035), potentially pathogenic bacteria decreased (*P* = 0.04), and abundances of SCFA-producing genera *Faecalibacterium* and *Prevotella* increased significantly. Other findings included a significant reduction of *CAG-352*, *Preveotellaceae_NK3B31-group*, and *Eubacterium coprostanoligenes* (group 2), and a trend of lowered Firmicutes-to-Bacteroidetes ratio (*P* = 0.067) and a reduction in *Ruminococcaceae* (group 3). Our findings suggest that a dietary intervention based on strawberries can positively alter the gut microbiota of healthy elderly people as seen in an enrichment of SCFA-producing genera, increased diversity, and a reduction in potentially pathogenic bacteria.

**IMPORTANCE:**

Aging is often associated with changes in the gut microbiome, including a decline in beneficial bacteria and an increase in potentially pathogenic species. Addressing these changes through lifestyle interventions is of significant interest. Our study demonstrates that a 10-week dietary intervention with strawberries can beneficially modulate gut microbial composition and diversity in healthy elderly individuals. Notably, the group consuming the highest amount of strawberries (without capers in olive oil) initially had higher abundances of potentially pathogenic bacteria. Here, the intervention led to increased abundances of the beneficial genera *Faecalibacterium* and *Prevotella*, which are linked to health benefits including reduced inflammation and improved lipid metabolism. These findings suggest that strawberry consumption can positively influence gut microbial composition, thereby contributing to overall health and disease prevention in older adults.

## INTRODUCTION

Targeting the gut microbiome to counteract aging-related loss of health in the elderly is a promising strategy due to the microbiomes’ interaction with nutrition, metabolism, and immunity, thereby acting as modifier of disease risk (1).

Generally, aging is defined as all the processes that lead to disease, dysfunction, and death, countering health and survival (2). A generic gut microbial feature of aging is the depletion of health-associated commensals and the enrichment of potential pathogens (1). For example, age-related loss of muscle mass and strength is associated with reduced *Bacteroides*, *Faecalibacterium*, and *Prevotella* abundances, and increased *Escherichia-Shigella* and *Klebsiella* abundances (3), and in hypertensive subjects, *Faecalibacterium prausnitzii* and *Ruminococcus hominis* are depleted compared to normotensive subjects (4).

Often, health-associated taxa are short-chain fatty acid (SCFA) producers, and thus potential biomarkers and therapeutic targets for a healthy gut ecosystem and metabolic health (5).

The SCFA butyrate is a key mediator of microbe–host cross talk (6), the primary energy source for colon cells, and crucial for maintaining gut barrier integrity, electrolyte and fluid absorption, and host metabolism; it further plays a role in immune modulation and aging, as butyrate limits inflammation (7, 8).

Importantly, the abundances of SCFA producers is influenced by dietary compounds including fibers and polyphenols present in fruits, vegetables, grains, seeds, nuts, and oils (9, 10). Substrates that are selectively utilized by host microorganisms and confer a health benefit are termed “prebiotics” (11). Polyphenols fall under this category because most of them are metabolized by the colonic microbiota, playing a central role in the bioavailability of polyphenols (11–13).

Polyphenols in plants serve as protective mechanisms against biotic and abiotic stresses; in humans, the consumption of polyphenol-rich foods has been linked to a lowered risk of chronic and degenerative diseases (14). In the MaPLE trial, a polyphenol-rich diet increased the abundances of butyrate producers, e.g., those of the *Faecalibacterium* genus*,* along with reducing blood pressure and intestinal permeability (15).

Of high-polyphenol foods, berries are especially popular and a dietary resource commonly consumed in many countries. Strawberries contain a plethora of distinct chemical compounds, including vitamins, minerals, fatty acids, and dietary fiber, and a range of polyphenols such as flavonoids (anthocyanins [pelargonidin, cyanidin], flavanols [catechin, epicatechins, procyanidins], and flavonols [quercetin, kaempferol, morin]); phenolic acids (hydroxybenzoic acids and hydroxycinnamic acids); and stilbenes (resveratrol) (http://phenol-explorer.eu/contents/food/69).

Several studies examined the effects of dietary supplementation with freeze-dried strawberries or strawberry polyphenols, and findings include improved learning and memory ability (16), improved insulin sensitivity (17), decreased levels of C-reactive protein (CRP), increased total antioxidant status (18), and increased abundances of beneficial taxa, including *Bifidobacterium* and *Akkermansia muciniphila* (19). However, the effects of a nutritional addition of varying amounts of strawberries in healthy elderly people otherwise consuming their habitual diet are underexplored.

## RESULTS

Group composition according to age and gender and the corresponding interventions are shown in Tables 1 and 2. There was no significant difference in age (*P* = 0.239) or ratio of females to males (*P* = 0.771) between the groups.

**TABLE 1:** Group composition and intervention schemes

Group	Group 1	Group 2	Group 3	Group 4	Group 5
*N*	12	14	15	11	17
Gender, F:M, *n*	7:5	10:4	11:4	9:2	11:6
Age in years (mean ± SD)	59.33 ± 5.09	61.57 ± 8.58	64.00 ± 7.68	64.64 ± 7.51	63.65 ± 5.74

**TABLE 2:** Consumption patterns of the high-polyphenol dietary intervention for each group and week.

Group	Dietary intervention	Week 1	Week 2	Weeks 3–9	Week 10
Group 1	Fresh strawberries	500 g	500 g	500 g	500 g
Group 2	Fresh strawberries	1,500 g	1,500 g	2,500 g	3,750 g
Group 3	Fresh strawberries	1,500 g	1,500 g	2,500 g	3,750 g
	Freeze-dried strawberries^*a*^	100 g	100 g	-	100 g
Group 4	Fresh strawberries	1,500 g	1,500 g	2,500 g	3,750 g
	Freeze-dried strawberries^*a*^	100 g	200 g	-	200 g
Group 5	Fresh strawberries	1,500 g	1,500 g	2,500 g	3,750 g
	Freeze-dried strawberries^*a*^	100 g	200 g	-	200 g
	Capers in olive oil^*a*^	120 g	240 g	-	240 g

^*a*^ On three single days. The amount and kind of intervention food varied according to group and week: group 1 served as the control group, consuming 500 g of fresh strawberries every week. The other groups had increasing quantities of intervention foods from weeks 1–2 to weeks 3–9 to week 10. The "-" refers to absence of the intervention food in that particular week.

### Community diversities

**Fig 1:**
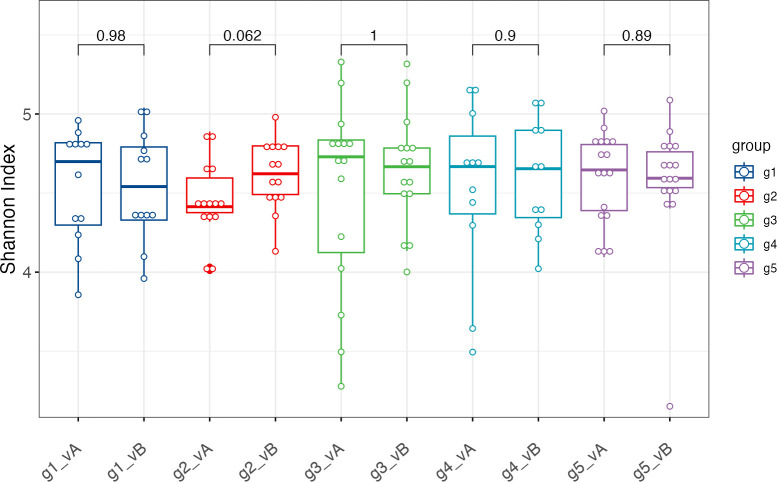
Shannon index to estimate the alpha diversities for each group (g1–g5) before (visit A, vA) and after (visit B, vB) the intervention. Differences between groups were assessed using the Kruskal-Wallis test, and differences between visits were assessed using the pairwise Wilcoxon test (20). Kruskal-Wallis *P* value = 0.98. Wilcoxon *P* values are denoted above the braces.

Differences in alpha diversity between groups and visits were assessed using the Shannon index. This index is based on both the number of species (richness) and how evenly the present species are distributed (evenness). In our study population, the range of per-group alpha diversities was between 3.15 and 5.33, with a mean index of 4.56. No single group had a significantly higher or lower index than the other groups after the intervention (Fig. 1, Kruskal-Wallis [KW] *P* value = 0.98) or at baseline (KW *P* value = 0.76).

**Fig 2:**
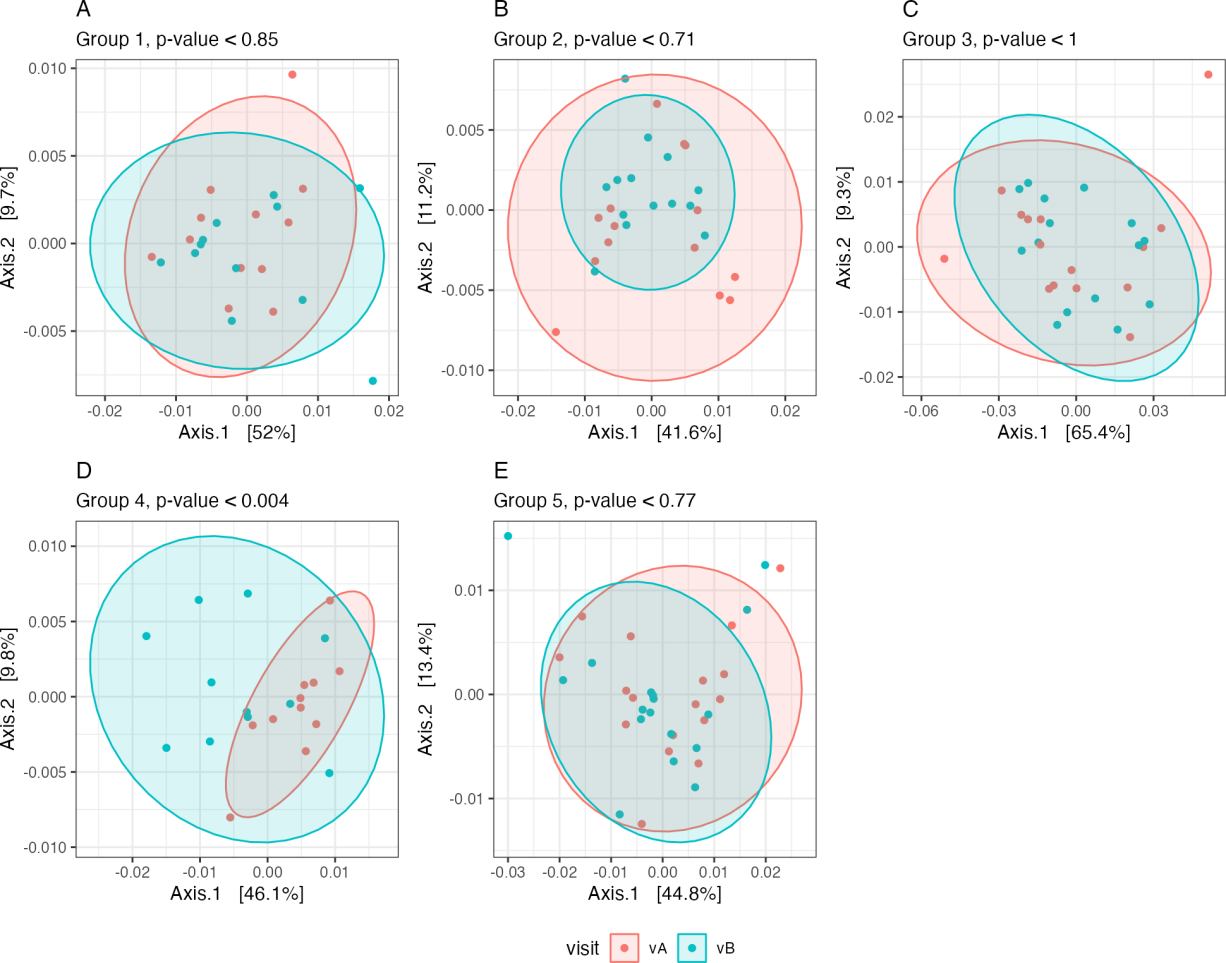
Beta diversity. The weighted UniFrac distance metric was used to estimate the dissimilarities between the samples of each group (A–E, groups 1–5) before (vA, red) and after (vB, blue) the intervention. Significance between visits was assessed with a PERMANOVA test (21).

Beta diversity is commonly used to identify differences in microbiome profiles between two conditions or groups. Here, we used weighted and unweighted UniFrac distances plotted by a principal coordinate analysis to visualize divergence between visits. The unweighted UniFrac distance considers the presence or absence of microbial taxa without regard to their abundances, whereas the weighted distance takes into account both the phylogenetic relationship among taxa and their relative abundances. Unweighted UniFrac distances showed no difference between visits, suggesting that the composition of microbial taxa remained unchanged. However, the weighted UniFrac distances showed significant differences in clustering for samples from group 4 (Fig. 2, permutational multivariate analysis of variance [PERMANOVA] *P* < 0.004), indicating that the relative abundances of the present taxa shifted significantly between the two visits.

### Variations in community composition between groups and visits

#### Phyla and F/B ratios

**Fig 3:**
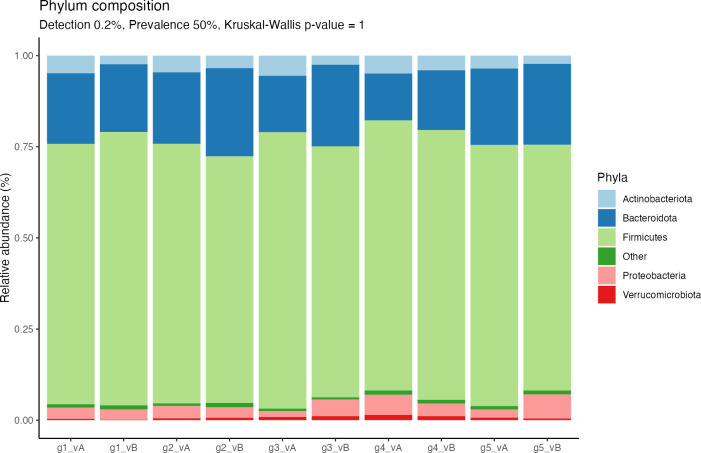
Phylum composition. Phyla with a prevalence of ≥50% across samples are included in the bar plot (22). For each group (g1–g5), the phylum composition was given before (visit A, vA) and after (visit B, vB) the 10-week intervention. The relative abundances of phyla between visits and groups were not significantly different (KW *P* values: vA = 0.98, vB = 0.99)*.*

**Fig 4:**
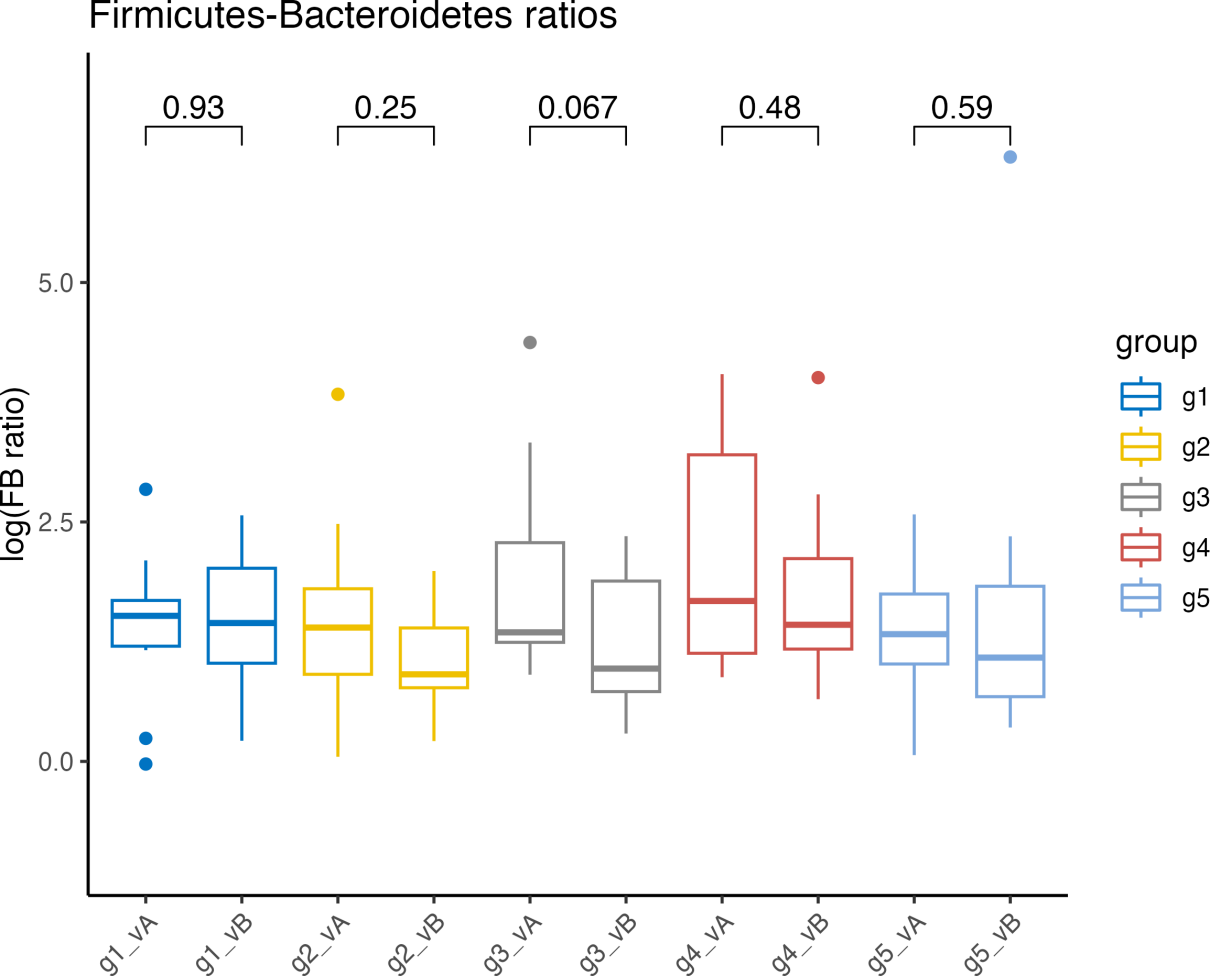
Firmicutes-to-Bacteroidetes ratios. For each group (g1–g5), the F/B ratios were given before (visit A, vA) and after (visit B, vB) the 10-week intervention. The significance between visits was assessed with a pairwise Wilcoxon test (20). *P* values are denoted above the braces.

Observed variations in phylum composition included higher relative abundances of Bacteroidetes in groups 2–5 and lower abundances of Firmicutes in groups 2, 3, and 5 after the intervention (Fig. 3; Table S1). In group 3, there was a trend of a lowered Firmicutes-to-Bacteroidetes (F/B) ratio (*P =* 0.067*),* while the other groups showed a reduction in mean F/B ratio (Fig. 4). In groups 3 and 5, Proteobacteria relative abundances increased but remained roughly the same in groups 1 and 2, and were lower in group 4 after the intervention. Of note, at baseline, group 4 was the group with the highest F/B ratio and the lowest relative abundance of Bacteroidetes.

#### Genera and core microbiomes

**Fig 5:**
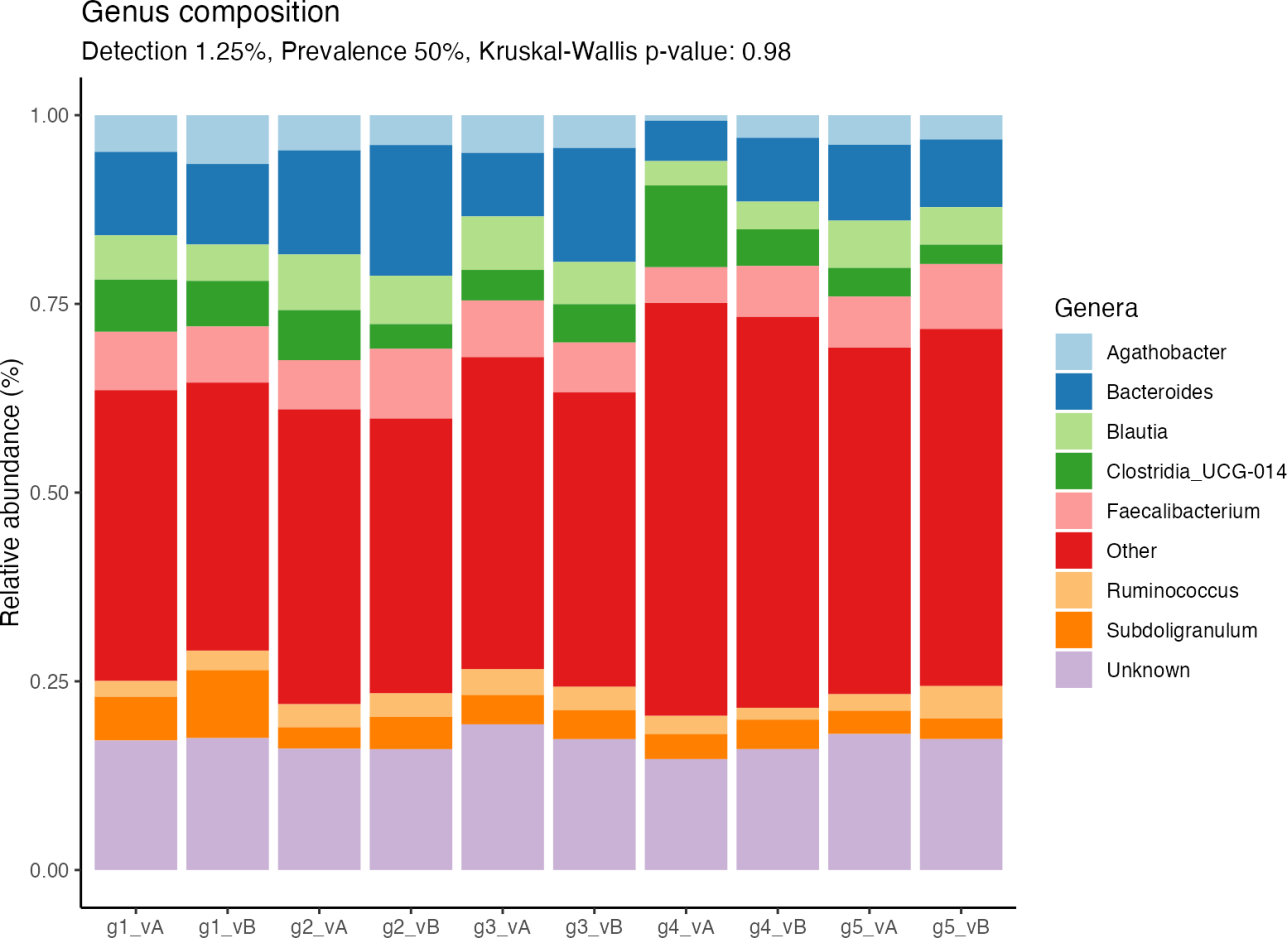
Genus relative abundances. Genera that were prevalent in at least half the samples and detected at ≥1.25% relative abundances are included in the barplot. For each group (g1–g5), the genus composition was given before (visit A, vA) and after (visit B, vB) the 10-week intervention.

There were variations in the composition of prevalent genera (≥50% of samples) between groups and visits (Fig. 5; Table S2), including *Bacteroides, Blautia, Agathobacter, Clostridia UCG-014, Faecalibacterium, Ruminococcus*, and *Subdoligranulum.* Some shared changes included higher *Bacteroides* abundances in groups 2, 3, and 4, and lower *Chlostridia UCG-014* abundances in groups 2 and 4 after the intervention. *Faecalibacterium* relative abundances increased in groups 2, 4, and 5.

**Fig 6:**
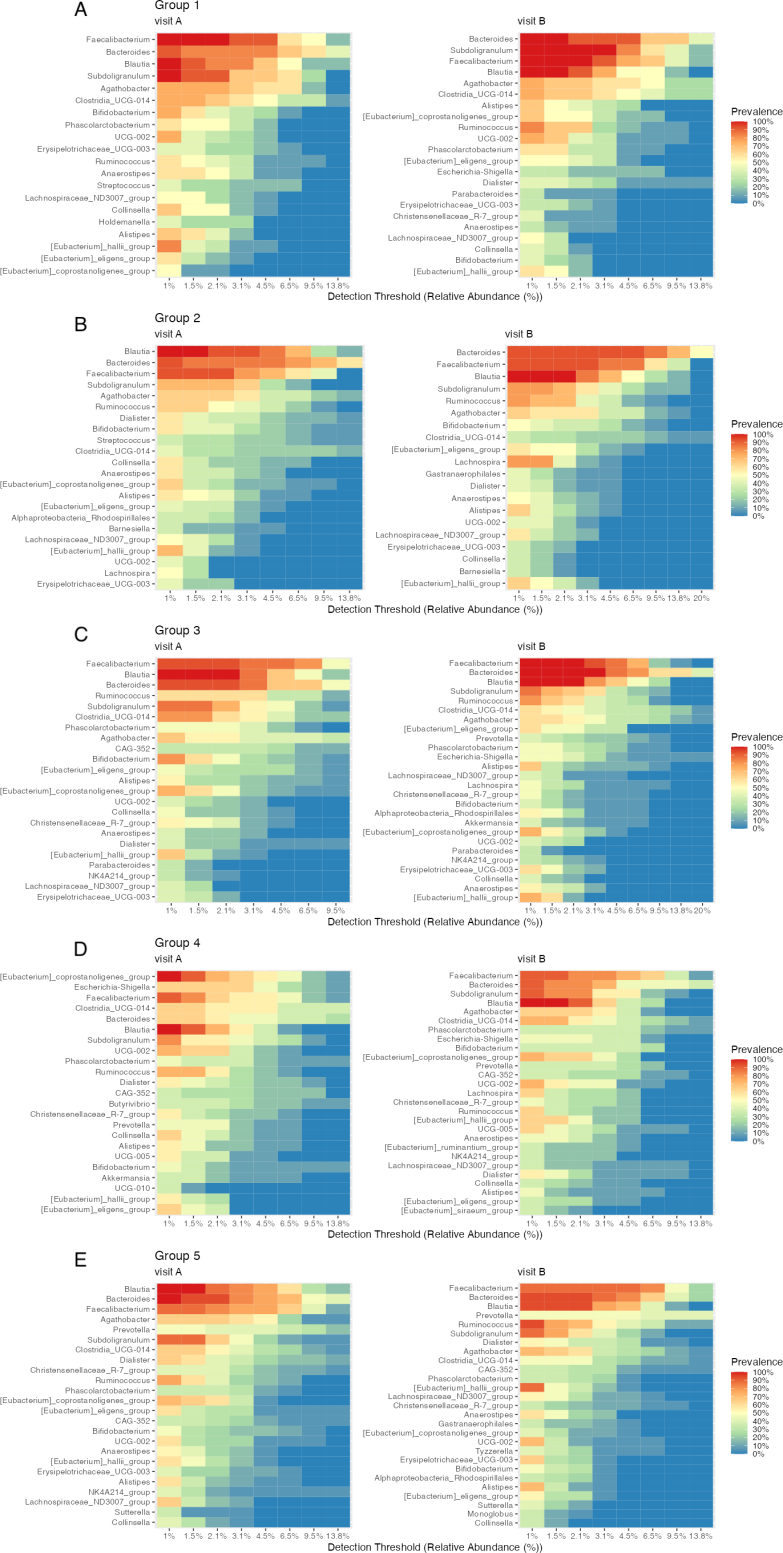
Core microbiomes of genera. Heatmaps A–E show the core genera of each group (g1–g5) with a prevalence of at least 30%. For each taxon, the colored fields correspond to the prevalence at a certain detection threshold and relative abundance. Left heatmaps (visit A) show the core genera before the intervention; right heatmaps (visit B) show the core genera after the intervention.

**Fig 7:**
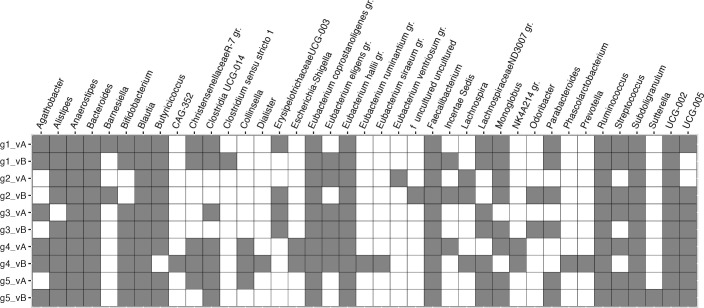
Comparison of core microbiomes. Of each group (g1–g5) and visit (vA, vB), genera with a prevalence of at least 80% and detected with at least 0.1% relative abundance are included in the heatmap.

Additionally, we plotted the core microbiomes of each group before and after the intervention in heatmaps (Fig. 6), showing the prevalence and detection levels of each taxon present in at least 30% of samples per group and visit, and the less-abundant genera that are not pictured in the genus abundance barplot, most of which were categorized into “Others” (Fig. 5). As microbiomes undergo temporal and seasonal variations, variations were also observed in every group including the control group. The strongest variation in core members was seen in group 4. At baseline, *E. coprostanoligenes, Escherichia-Shigella, Faecalibacterium*, and *Clostridia-UCG-014* were the most prevalent genera with the highest relative abundances across samples. After the intervention, the composition of core members was more similar to the core microbiomes of groups 1, 2, and 3 (before and after the intervention), with the highest prevalence and abundance of *Faecalibacterium, Bacteroides, Blautia*, and *Subdoligranulum.* Additionally, highly prevalent genera (prevalence >80% of each group and visit) were plotted in a presence/absence heatmap (Fig. 7). A higher prevalence of *Escherichia-Shigella* was unique to group 4 at the first but not at the second visit. The genus *Sutterella* was uniquely detected in group 5 after the intervention.

### Phenotypic changes

**Fig 8:**
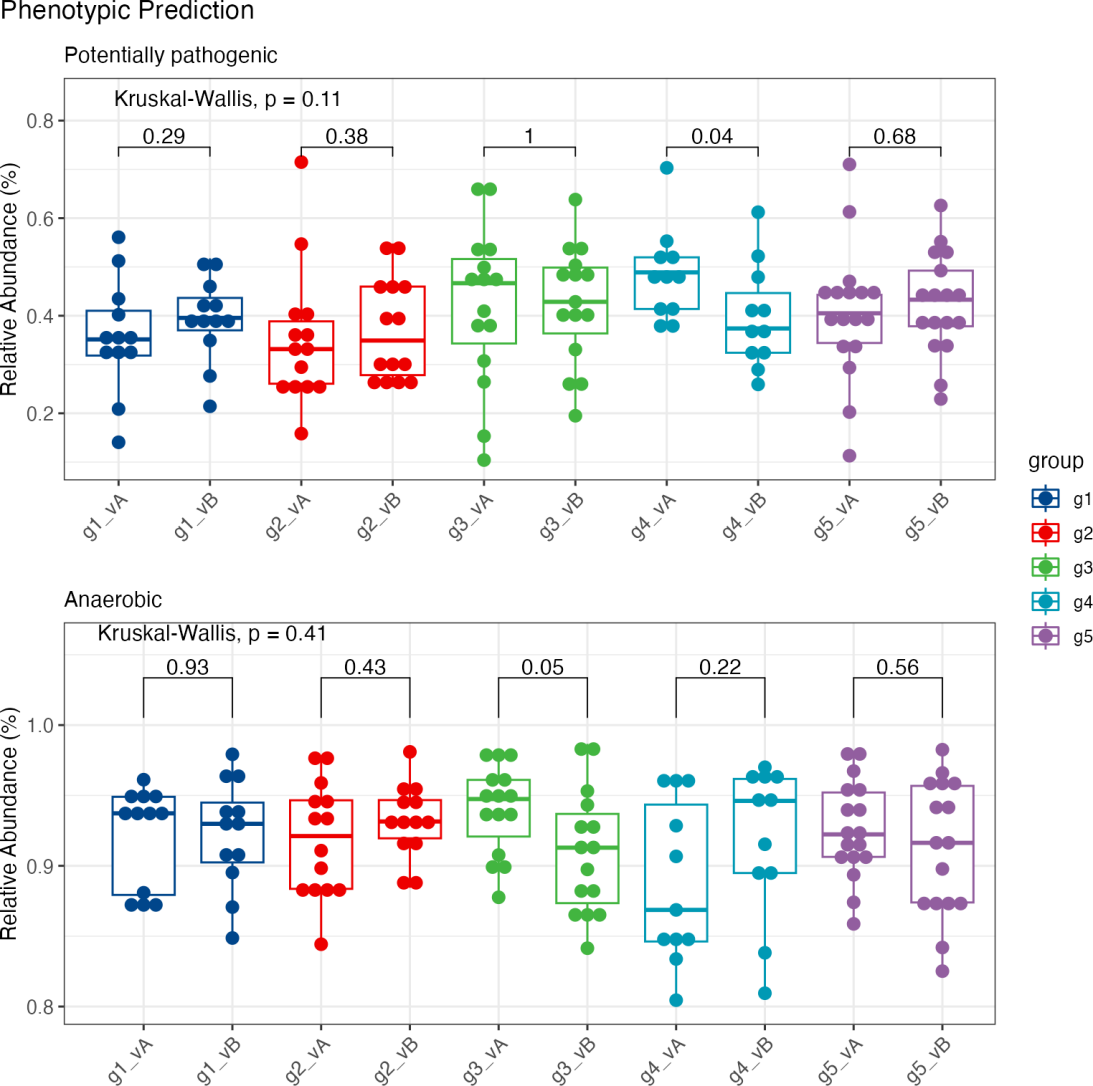
Phenotypic prediction via BugBase (23), showing the plots with significant differences in phenotypes. The significance between visits was assessed using a Wilcoxon test.

We utilized the BugBase algorithm to infer biological phenotypes, as described by Ward et al. (23). Notable alterations in phenotypes were found in groups 3 and 4 post-intervention (Fig. 8; Table S3). In group 3, the relative abundance of anaerobic bacteria was lower (*P* = 0.05). Importantly, in group 4, potentially pathogenic bacteria decreased, where mean abundances of 48% before the intervention were lowered to 37% relative abundance after the intervention (*P* = 0.04), which could be attributed to lowered abundances of *Escherichia-Shigella*. Additionally, the relative abundance of bacteria containing mobile elements was lower in group 3 (KW *P* = 0.036) than in group 1 (*q* = 0.0008) and group 4 (*q* = 0.0088) (not shown).

### Differentially abundant taxa and pathways

To investigate the differences between the two visits (after the intervention vs before the intervention) on the taxon level, we carried out a differential abundance testing using DESeq2. We performed this analysis for all samples per group as well as combining all samples from intervention groups 2–5.

We found significant differences in abundances of genera between the visits in all groups (Table 3) except in the highest intervention group, group 5, but we did not find such differences between visits when considering all samples of groups 2–5 together. Of note, two differentially abundant genera were SCFA producers, *Faecalibacterium* and *Prevotella,* which increased in abundance in group 4.

**TABLE 3:** Taxa with significant changes in abundance after the strawberry intervention, with false discovery rate (FDR) < 0.05^*a*^

Group	Taxon	Change	FDR	SCFA
Group 1	*Clostridia UCG-014*	Down	<1e-10	
	*Clostridia UCG-014*	Down	<1e-10	
Group 2	*Ruminococcaceae*, *CAG-352*	Down	<1e-11	
	*Prevotellaceae_NK3B31_group*	Down	<1e-11	
	*Eubacterium coprostanoligenes*	Down	<1e-11	
Group 3	*Ruminococcaceae*	Down	<1e-11	
Group 4	*Faecalibacterium*	Up	<1e-9	Butyrate (24)
	*Prevotella*	Up	<1e-9	Propionate (25)

^*a*^ The “change” column refers to whether the taxon was found in decreased (down) or increased (up) abundances after the intervention. The “SCFA” column refers to the metabolite or metabolites (i.e., acetate, propionate, and/or butyrate) produced by the differentially expressed taxon.

We also used PICRUSt1 to predict potential functional changes of the intervention. Five pathways were enriched between visits, but the changes were not significant when *P* values were false-discovery corrected. In group 3, these were lipoic acid metabolism (*P =* 0.0038, *q* = 0.48), ubiquinone and other terpenoid-quinone biosynthesis (*P =* 0.0090, *q* = 0.48), and membrane and intracellular structural molecules (*P =* 0.0092, *q* = 0.48). In group 4, these were phenylpropanoid synthesis (*P =* 0.0048, *q* = 0.36) and cyanoamino acid metabolism (*P =* 0.0056, *q* = 0.36).

## DISCUSSION

Our results show that a dietary intervention rich in strawberries, over a period of 10 weeks, can lead to changes in diversity and composition.

The gut microbial profile observed in group 4 at baseline was characterized by high abundances of *Clostridia-UCG-014* and *Escherichia-Shigella* and low abundances of *Faecalibacterium*, *Bacteroides*, and *Agathobacter*. This group comprised the dietary intervention with the highest consumption of strawberries (without capers in olive oil). After the intervention, beta diversity was significantly different compared to baseline, reflecting the shift in abundances of the present taxa. *Faecalibacterium* and *Prevotella* were enriched after the intervention. Both genera are key SCFA producers that typically decline in abundance with age (26) and in disease. For example, *Faecalibacterium* is often depleted in metabolic syndrome, Crohn’s disease (27), multiple sclerosis, arthritis (28), certain cancers (29, 30), coronavirus disease 2019 (COVID-19) (31), depression (32), and Parkinson’s disease (33).

Among the physiological functions of SCFAs, the regulation of inflammation and lipid metabolism are of special relevance. Previously, it was found that low-density lipoprotein (LDL) cholesterol and CRP levels were significantly reduced in high-intervention participants of the ErdBEHR study, as compared to the control group (34). Specifically, the intervention with fresh strawberries resulted in a decrease in LDL by 0.0174 mmol/L per 500 g of fresh strawberries consumed weekly (34).

For a long time, studies have shown that the addition of fiber to the diet reduces cholesterol, and the benefits have been attributed to SCFA-producing bacteria that selectively ferment these substrates to the main SCFAs acetate, propionate, and butyrate (35, 36). This effect may have been synergistic with any more direct effects of the polyphenols themselves(37).

Blood cholesterol, in particular higher LDL and lower high-density lipoprotein (HDL) concentrations, indicate a higher probability of atherosclerotic plaque development and atherosclerosis (38), which is the most common form of cardiovascular disease. Atherosclerosis is characterized by lipid accumulation and chronic inflammation of the large arteries, and links between the gut microbiome and atherosclerosis are being increasingly recognized (39). For example, a metagenome-wide association study indicated differences in gut microbial composition between atherosclerotic cardiovascular disease patients and healthy controls, where *Bacteroides* and *Prevotella* as well as butyrate producers such as *Faecalibacterium prausnitzii* and *Roseburia intestinalis* were relatively depleted compared to healthy controls, and *Streptococcus* and *Escherichia* were enriched (40). In a study conducted in apolipoprotein E-deficient mice, butyrate limited cholesterol uptake in a dose-dependent manner with less atherosclerotic lesions, suggesting that butyrate protects from diet-induced atherosclerosis (41).

Additionally, as there was a decrease in CRP in the main study, the associations of CRP/inflammation and SCFAs are of interest. Generally, CRP production is induced by proinflammatory cytokines that activate kinases and phosphatases leading to translocation of transcription factors and to its production of CRP (42). Even a minor elevation in baseline CRP concentration is linked to low-grade inflammation and is therefore an important underlying factor in many common diseases (42). The anti-inflammatory role of butyrate is partly due to its inhibitory effect on NF-kB (43), which is important in diseases characterized by chronic low-grade inflammation (44). Inhibition of NF-kB modulates proinflammatory cytokine production, such as inhibition of IL-12 and IFN-γ secretion (45). SCFAs also increase anti-inflammatory IL-10 production via direct regulation of T-cell differentiation through suppression of histone deacetylase (HDAC) activity in CD4+ T-cells (46). Butyrate can also indirectly induce IL-10-positive T-cells via binding to *free fatty acid receptor 3* on macrophages and dendritic cells (47), with anti-inflammatory effects.

Given the anti-inflammatory properties of SCFAs, the increase of SCFA-producing bacteria may have contributed to the observed CRP-lowering effect of the strawberry dietary intervention. Taken together, this finding suggests that strawberries may support the maintenance of a healthy gut microbiome in older adults.

Besides, it is important to acknowledge the limitations of our study. The small size of the different groups of participants (11–17 people) and the compositional and dynamic nature of microbiomes limited the statistical power to detect significant changes. Moreover, nutritional interventions in healthy individuals typically do not produce strong signals. Thus, the most robust findings are those supported by several pieces of evidence; in particular, the increase in SCFA-producing bacteria stands out, given their anti-inflammatory effect, which was specifically corroborated by the reduction of inflammation in group 4 in the main study. Additionally, although the study was part of a randomized, controlled trial, the donation of fecal samples was optional and the people who donated samples were self-selected.

To conclude, our results, especially in the group with the highest strawberry intake without capers in olive oil, group 4, indicate that a dietary intervention incorporating strawberries can promote a beneficial shift in the gut microbiomes of older adults. This shift, characterized by increased beta diversity and higher abundances of health-related SCFA-producing genera, suggests a positive impact on gut microbiome structure and composition. These findings contribute to the growing body of evidence emphasizing the role of diet in maintaining and promoting health, particularly in aging populations.

## MATERIALS AND METHODS

Here, we describe the ErdBEHR nutritional intervention trial, focusing on relevant aspects of our investigation of the gut microbiome. For a full account, see Hartmann et al. (34). The ErdBEHR study took place in north-east Germany in summer, from 10 June to 1 October 2021. A total of 168 participants were randomly assigned to one of five groups and were instructed to eat different amounts of strawberries with or without additional polyphenol-rich foods (freeze-dried strawberries with or without capers in olive oil) for 10 weeks.

In addition to blood samples, 70 out of 168 study participants agreed to provide fecal samples. Sixty-nine participants provided samples at two timepoints, before and after the intervention period, here referred to as “visit A” and “visit B.” The average age of participants was 62.7 years, and 69.5% were female. Eligible age for participation was between 50 and 80 years. Other inclusion criteria were an intake of dietary supplements or vitamins not exceeding standard recommendations and a weight of less than 100 kg. Participants had to have access to a strawberry salespoint to obtain fresh fruit regularly. Subjects with known food intolerances/allergy to strawberries were excluded from the study, as well as those with severe chronic diseases and participants taking prescription drugs except cholesterol-lowering agents, anti-hypertensive drugs, and thyroid medications.

### Intervention groups

Group 1 was the control group and was asked to consume 500 g of strawberries per week for the duration of the study (10 weeks). Intervention groups 2–5 consumed 1,500 g of strawberries per week in weeks 1 and 2, 2,500 g of strawberries in weeks 3–9, and 3,750 g of strawberries in week 10. Intervention groups 3, 4, and 5 also featured freeze-dried strawberries in the dietary intervention, with 100 g of freeze-dried strawberries to be eaten on the first day of the study for groups 3, 4, and 5. In group 3, 100 g of freeze-dried strawberries was also provided to be eaten on days 14 and 69. Similarly, groups 4 and 5 were asked to eat 200 g of freeze-dried strawberries on days 14 and 69. Group 5 was the highest intervention group and, in addition to the fresh and freeze-dried strawberries as in group 4, capers in olive oil were included: 120 g on day 1, and 240 g on days 14 and 69. Participants were asked to consume these extra dietary components on three single days, two of these days in the 2 weeks after the first microbiome sampling, and the last one on the day before the second microbiome sampling.

The participants were instructed to follow the intervention plan, but only if the food was not causing a high degree of aversion; in this case, participants were instructed to inform the study personnel at the next visit. Further instructions included to refrain from cooking or baking the foods, to consume the olive oil together with the capers, to otherwise stick to their normal dietary habits, and to not eat foods with a specific health-related effect regularly or in high amounts (mentioning green tea or garlic as examples).

### Randomization and blinding

The randomized group allocation was not revealed to the involved physicians and laboratory personnel. The participants were not blinded to groups.

### Sampling and DNA extraction

DNA was isolated from stool samples with the QIAamp Fast DNA Stool Mini Kit (Qiagen, Hilden, Germany) according to the instructions of the manufacturer.

### Amplicon library preparation and sequencing

The 16S rRNA V3 and V4 variable regions were amplified via polymerase chain reaction with forward primers 341F (TCGTCGGCAGCGTCAGATGTGTATAAGAGAC AGCCTACGGGNGGCWGCAG) and reverse primers 805R (GTCTCGTGGGCTCGGAGATGTGTATAAGAGACAGGACTACHVGGGTATCTAATCC), additionally containing sequencing adapters and a single-end barcode (reverse primer), allowing for pooling and direct sequencing of resulting amplicons. A PhiX control library (Illumina) was combined with the amplicon library (expected at 20%). The prepared libraries were sequenced in 300 paired-end MiSeq runs. The sequencing data met the quality requirements, with an average Q30 score exceeding 75% (indicating high-quality bases) and a minimum of 25,000 filtered reads per sample. On average, each sample yielded between 50,000 and 60,000 reads. The image analysis, base calling, and data quality assessment were performed on the MiSeq instrument.

Sequencing was carried out by Biovis Diagnostik, Limburg-Offenheim, Germany. Microbial paired-end sequences were processed with QIIME2 (v.2-2022.2) (48). Sequences were demultiplexed via “demux” and were denoised via “denoise-16S.” For taxonomic assignment, we used SILVA database version 138-99 (49).

### Statistical analysis

Microbiota operational taxonomic unit (OTU) reads were imported into R 4.2.1 where statistical analyses were carried out with the phyloseq (v.1.40.0) (50) and vegan (v.2.6-4) packages (51). Significant differences between groups were tested using the Kruskal-Wallis, and significant differences between visits were tested using the Wilcoxon rank-sum test (20) unless otherwise stated.

The microbiome (v. 1.18.0) package (22) was used to assess phylum and genus community compositions and core microbiomes. Core genera were visualized with a heatmap using the “plot_core” function (22). For each group and visit, genera below a prevalence of 30% were filtered out. To identify which genera were shared between groups and visits, highly prevalent genera detected with at least 0.1% relative abundance and at least 80% prevalence were included in a presence/absence heatmap.

Alpha diversity was quantified with the Shannon index using the “estimate_richness” function from phyloseq. Between-group diversity (beta diversity) was assessed using weighted and unweighted UniFrac-distances (52). Here, we carried out a PERMANOVA with 8,000 permutations to test for significance (21).

The ratio between Firmicutes and Bacteroidetes was determined by dividing the relative abundances of the two phyla of each group at both time points. Differentially abundant taxa were assessed via the Negative Binomial Wald Test from the DESeq2 package (v.1.36.0) with the “Wald” test and “parametric” fit type (53). PICRUSt 1.1.4 was used for metagenome predictions, i.e., to find pathways associated with the taxa, and DESeq2 was used to identify differentially abundant pathways with the “Wald” test and “parametric” fit type. A false discovery rate (FDR)-adjusted *P* value (*q* value) of less than 0.05 was used to determine significance.

Phenotypic prediction/characterization was carried out with the BugBase algorithm (23). For this, the reads were aligned to the GreenGenes database (version 13_8) based on a 97% OTU similarity threshold using QIIME2. The corresponding OTU table was uploaded to the BugBase server (https://bugbase.cs.umn.edu/upload.html) to obtain predictions.

## Data Availability

The 16S rRNA sequencing data generated and analyzed during this study has been deposited in the European Nucleotide Archive under accession number PRJEB83578 .

## SUPPLEMENTAL MATERIAL

Tables S1 to S3 spectrum.01913-24-s0001.pdf Contains tables of phylum and genus relative abundances, and significant differences in phenotypic predictions.

ASM does not own the copyrights to Supplemental Material that may be linked to, or accessed through, an article. The authors have granted ASM a non-exclusive, world-wide license to publish the Supplemental Material files. Please contact the corresponding author directly for reuse.
