# Supplementary material for: Strawberry dietary intervention influences diversity and increases abundances of SCFA-producing bacteria in healthy elderly people
Source: Microbiol Spectr. 2025 Jan 8;13(2):e01913-24. doi: 10.1128/spectrum.01913-24 (PMC11792484; doi:10.1128/spectrum.01913-24)
Supplement: Tables S1 to S3 — Contains tables of phylum and genus relative abundances, and significant differences in phenotypic predictions. [file spectrum.01913-24-s0001.pdf]

Strawberry Dietary Intervention Influences Diversity and Increases Abundances of SCFA Producing Bacteria in Healthy Elderly People

Franziska Meiners[1], Bernd Kreikemeyer[2], Patrick Newels[3], Ingmar Zude[3], Michael Walter[4], Alexander Hartmann[4], Daniel Palmer[1], Georg Fuellen[1,5*], Israel Barrantes[1]

[1] Institut für Biostatistik und Informatik in Medizin und Alternsforschung, Universitätsmedizin Rostock, Rostock, Germany

[2] Institut für Medizinische Mikrobiologie, Virologie und Hygiene, Universitätsmedizin Rostock, Rostock, Germany

[3] Biovis Diagnostik, Limburg-Offenheim, Germany

[4] Institut für Klinische Chemie und Laboratoriumsmedizin, Universitätsmedizin Rostock, Rostock, Germany

[5] Conway Institute of Biomolecular and Biomedical Research, School of Medicine, University College Dublin, Dublin, Ireland

*Corresponding author

Number of Supplementary Tables: 3

Supplementary Table Legends

Table S1: Phyla in the study population for each group (g1-g5) and visit (vA, vB) with a prevalence of at least 30% and 1% relative abundance, and F/B ratio.

Table S2: Genus relative abundances of core taxa of the study population for each group (g1-g5) and visit (vA, vB). Prevalence of at least 70%, relative abundance > 0.5%.

Table S3: Significant differences in relative abundances of phenotypic predictions identified using the BugBase algorithm (Ward et al., 2017). g = group. Significance was assessed using a Wilcoxon test.

Supplementary Tables

Table S1: Phyla in the study population for each group (g1-g5) and visit (vA, vB) with a prevalence of at least 30% and 1% relative abundance, and F/B ratio.

Phylum	g1_vA	g1_vB	g2_vA	g2_vB	g3_vA	g3_vB	g4_vA	g4_vB	g5_vA	g5_vB
**Firmicutes**	71.42%	74.96%	71.20%	67.63%	75.81%	68.81%	74.07%	73.98%	71.58%	67.39%
**Bacteroidetes**	19.37%	18.58%	19.62%	24.19%	15.48%	22.37%	12.84%	16.41%	20.99%	22.17%
**Actinobacteriota**	4.84%	2.36%	4.58%	3.43%	5.53%	2.51%	4.91%	4.02%	3.53%	2.26%
**Proteobacteria**	3.05%	2.81%	3.36%	2.86%	1.57%	4.58%	5.50%	3.43%	2.23%	6.58%
**Verrucomicrobiota**	0.37%	0.11%	0.50%	0.66%	0.88%	1.10%	1.45%	1.09%	0.71%	0.47%
**Cyanobacteria**	0.83%	1.00%	0.53%	0.82%	0.62%	0.51%	0.99%	0.71%	0.44%	0.84%
**Desulfobacterota**	0.11%	0.16%	0.15%	0.17%	0.07%	0.09%	0.18%	0.14%	0.21%	0.19%
**F/B ratio**	3.69	4.03	3.63	2.80	4.90	3.08	5.77	4.51	3.41	3.04

Table S2: Genus relative abundances of core taxa of the study population for each group (g1-g5) and visit (vA, vB). Prevalence of at least 70%, relative abundance > 0.5%.

Genus	g1_vA	g1_vB	g2_vA	g2_vB	g3_vA	g3_vB	g4_vA	g4_vB	g5_vA	g5_vB
**[Eubacterium]_coprostanoligenes_group**	0.83%	2.1%	1.84%	0.76%	1.9%	1.4%	5.24%	2.5%	1.9%	1.1%
**[Eubacterium]_hallii_group**	1.48%	1.1%	1.17%	1.50%	1.1%	1.3%	1.03%	2.0%	1.4%	1.6%
**Agathobacter**	4.86%	6.5%	4.66%	3.96%	5.0%	4.3%	0.73%	3.0%	3.9%	3.2%
**Alistipes**	1.45%	2.1%	1.72%	1.54%	1.8%	1.9%	1.44%	1.1%	1.3%	1.0%
**Bacteroides**	**11.05%**	**10.7%**	**13.79%**	**17.32%**	**8.4%**	**15.1%**	**5.35%**	**8.4%**	**10.1%**	**9.0%**
**Blautia**	5.88%	4.8%	7.38%	6.40%	7.1%	5.6%	3.23%	3.7%	6.3%	5.0%
**Faecalibacterium**	**7.74%**	**7.4%**	**6.52%**	**9.24%**	**7.5%**	**6.6%**	**4.75%**	**6.8%**	**6.8%**	**8.6%**
**Ruminococcus**	2.12%	2.6%	3.08%	3.14%	3.4%	3.1%	2.43%	1.6%	2.2%	4.3%
**Subdoligranulum**	**5.79%**	**9.0%**	**2.82%**	**4.28%**	**3.9%**	**3.9%**	**3.32%**	**3.9%**	**3.1%**	**2.7%**
**UCG-002**	1.78%	2.5%	0.76%	1.08%	1.0%	1.0%	2.44%	2.1%	1.6%	1.4%
Other	39.87%	33.7%	40.17%	34.76%	39.5%	38.4%	55.34%	48.9%	43.5%	44.8%
Unknown	17.16%	17.5%	16.09%	16.01%	19.3%	17.3%	14.70%	16.0%	18.0%	17.3%

Table S3: Significant differences in relative abundances of phenotypic predictions identified using the BugBase algorithm (Ward et al., 2017). g = group. Significance was assessed using a Wilcoxon test.

**Phenotypes**	**group**	**change**	**visit 1 (median)**	**visit 2 (median)**	***p*-value**
Anaerobic	g3	↓	0.947	0.912	0.05
Gram_Negative	g3	↑	0.118	0.186	0.05
Gram_Positive	g3	↓	0.881	0.813	0.05
Potentially_Pathogenic	g4	↓	0.488	0.373	0.04
